# Feasibility and Preliminary Effectiveness of the Homework Intervention Strategy (eHIS) Program to Enhance Male Condom Use: Research Protocol

**DOI:** 10.2196/resprot.7937

**Published:** 2018-01-02

**Authors:** Marta Glowacka, Lucy Yardley, Nicole Stone, Cynthia A Graham

**Affiliations:** ^1^ Department of Psychology Faculty of Social, Human and Mathematical Sciences University of Southampton Southampton United Kingdom

**Keywords:** condoms, pleasure, telemedicine, eHealth, risk reduction behavior, sexual behavior, health psychology, behavior modification

## Abstract

**Background:**

Although condoms are effective in reducing the risk of sexually transmitted infections (STIs) and unintended pregnancy, they are still often not used consistently and correctly. Negative impact on sensation and pleasure, ruining the mood, causing problems with maintaining erection, and condom slippage or breakage are some of the reasons given by men explaining why they do not want to use condoms. Although many interventions promoting condom use exist, some of them delivered online are complex and time- and resource-intensive. The Homework Intervention Strategy (eHIS) program, adapted from the existing face-to-face Kinsey Institute Homework Intervention Strategy (KIHIS) program, aims to address these issues by encouraging men to focus on sensation and pleasure when trying different types of condoms and lubricants in a low-pressure situation (on their own, without a partner present).

**Objective:**

The objectives of this study are to assess the feasibility, acceptability, and users’ engagement with the eHIS program, its preliminary effectiveness in increasing condom use frequency and consistency, as well as the feasibility of the program's evaluation approach, including choice of measures and participant recruitment and retaining strategies (primary outcomes). Secondary outcomes include condom use experience, condom use attitudes, condom use self-efficacy, condom use errors and problems, and condom fit-and-feel. All of these will be analyzed in the context of participants’ demographics, sexual history, and previous condom use.

**Methods:**

The study has a pre-post-test, within-subjects design. Men aged 18 to 69 and living in the United Kingdom are recruited through posters, leaflets, social media, and emails. Study participants are asked to complete T1 (baseline) measures before entering the eHIS website. After completing the T1 measures, they can order a free condoms and lubricants kit and have access to the eHIS website for 4 weeks. During that time they are asked to practice using different types of condoms and lubricants on their own in a no-pressure situation. Following T1, participants are asked to complete the T2 and T3 measures at 4 and 10 weeks, respectively.

**Results:**

Data collection for the study is completed. Data analysis is in progress and is expected to be completed by February 2018.

**Conclusions:**

This brief, home-based, self-guided program may lead to increased consistent and correct condom use. Online delivery can make the program an easily accessible and low-cost health promotion intervention, which has the potential to reach a wide and diverse audience. If results of the current study show the program’s feasibility and preliminary effectiveness in changing condom use related outcomes, a larger scale randomized controlled trial (RCT) will be conducted.

**Trial Registration:**

Research Registry: researchregistry2325; http://www.researchregistry.com/browse-the-registry.html# home/registrationdetails/58da6cad1d7ab0314337d076/ (Archived by WebCite at http://www.webcitation.org/6vXs6S9XW)

## Introduction

### Background

Male condoms remain the single best method of reducing the risk of acquiring sexually transmitted infections (STIs), including HIV [1,2]. Promotion of correct and consistent use of male condoms as an effective method of reducing the prevalence of STIs was recommended in the Global Strategy for Prevention and Control of Sexually Transmitted Infections: 2006-2015 [3]. However, research repeatedly demonstrates that condoms are not used consistently [4-7], and even when used, condom use errors and problems and dislike of condoms are often reported [8,9]. Condom use errors and problems are associated with the reasons men give for not using condoms, such as less pleasurable experience when condoms are used, decreased sensation, poor fit-and-feel, condom breakage and slippage, and difficulties in maintaining erection [10,11].

Many previous interventions aimed to increase condom use but few of them focused on pleasure and fit-and-feel [12], and they were also often resource- and time-intensive. Improving fit-and-feel should lead to reduction in condom use problems and increase consistent and complete condom use. Internet-based behavior change interventions, on the other hand, may be cost- and resource-effective [13-15], easily accessible and acceptable by users, especially when focused on sensitive or stigmatized health-related issues [16-19], and have efficacy comparable with human-delivered interventions focused on condom use [20,21].

The Homework Intervention Strategy (eHIS) program, an online adaptation of the Kinsey Institute Homework Intervention Strategy (KIHIS) [22], combines a focus on pleasure and fit-and-feel in condom promotion with the benefits of an online intervention. In the KIHIS, during the session with the instructor, men are given the correct condom use instruction and are asked to practice condom application on a penile model. The instructor then encourages them to practice using condoms on their own and rate them, highlighting the importance of pleasure and finding the condom that fits and feels best for an individual. The home-based and practice-oriented approach makes the program distinct from most interventions in this area, which are mainly delivered face-to-face, during group workshops, or in individual consultations [23-25]. The results of previous pilot studies [22,26,27], showed the program’s potential in improving use experiences, confidence in the ability to use condoms, self-efficacy for condom use, condom comfort, and reduced breakage and erection problems.

The final content and design of the program was developed taking into account participant feedback from the qualitative evaluations of the program prototype (paper-based) and computerized version of the program (M Glowacka, thesis chapter in preparation). The research team adapting the face-to-face version of the program for use in the United Kingdom was also consulted [27].

**Figure 1:**
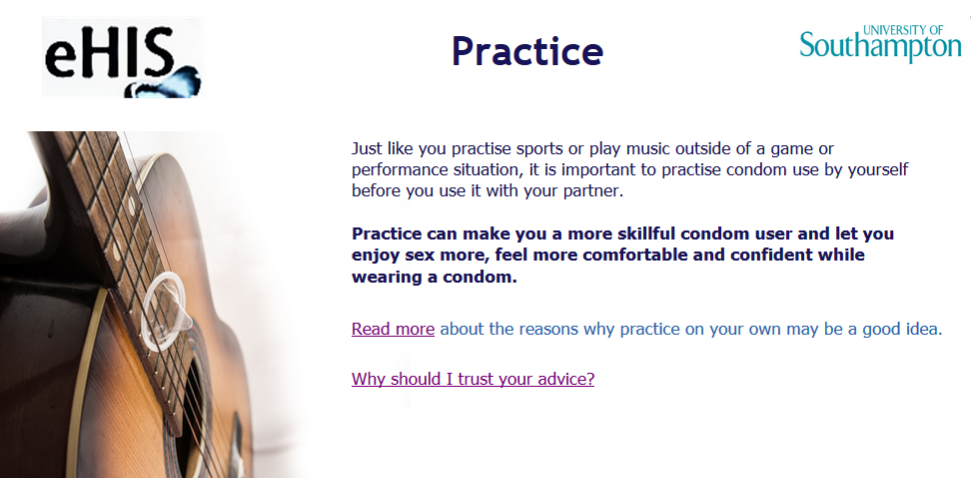
An example of a page on the Homework Intervention Strategy (eHIS) website.

Mirroring the KIHIS approach, the eHIS addresses the issues related to condom use errors and problems by focusing on correct condom use, pleasure, and developing positive condom use experience. Participants are encouraged to practice correct condom application and explore different types of condoms and lubricants in a low-pressure situation, at home, and without their partners present. Components of the intervention are listed in Textbox 1 and an example of the eHIS webpage is shown in Figure 1.

### Aims and Objectives

The current study aims to evaluate the feasibility of the Internet-based eHIS program. Evaluation of participants’ engagement with the program and its acceptability (dimensions of feasibility, primary outcomes) and the potential of the intervention to change targeted behavior (preliminary effectiveness) can provide a “proof of concept” for the approach used in the intervention [28,29]. The primary outcomes of this study are increasing condom use frequency and consistency. The secondary outcomes include reducing condom use errors and problems, enhancing condom use experience, increasing condom use self-efficacy, and improving condom use attitudes and motivation.

The study does not target men based on characteristics such as sexual orientation or condom use history as it has not yet been established for whom the eHIS intervention may be the most useful. Therefore, whether the program’s feasibility and preliminary effectiveness are linked to participants’ demographic characteristics, sexual history, or previous condom use variables is explored. To inform development of a larger trial, the feasibility of the approach to study evaluation with a focus on recruitment effectiveness, measures completion, and attrition rate is investigated. This will help to verify whether the specific study design and approach employed for the evaluation are appropriate and identifies acceptable outcome measures that should be used as measures of its effectiveness in a full-scale randomized controlled trial (RCT) [30]. The results can also help to estimate the expected effect size of the observed changes, to be used in the calculation of the sample size needed for a full scale RCT [31].

### Research Questions

This study is guided by the following research questions: (1) Is the eHIS program feasible? (2) Does the eHIS program have the potential to be effective in increasing the frequency of condom use, increasing consistent condom use, improving condom use experience, improving condom use self-efficacy, reducing the number of condom-related errors and problems, changing condom use attitudes to more positive ones, and increasing motivation to use condoms? (3) Is the approach to evaluate the eHIS program feasible? (4) Are the program and study feasibility, and the preliminary effectiveness of the program on condom use outcomes associated with participants’ characteristics (demographic, sexual history or baseline condom use variables)?

**The components of the Homework Intervention Strategy (eHIS) program.**

Core pages

- Program's rationale
- Correct condom use skills review
- Tips on how to deal with specific condom use errors and problems
- Information about program procedure and condom kit content
- A home practice guide
- Condom rating forms
- Ratings feedback
- Condoms and lubricants kit order

Optional pages

- Masturbation
- Partner involvement
- Condom effectiveness
- Information where to find support in case of concerns related to condom use
- Condom use instruction in various formats
- Example of condom rating form
- Motivational message (aimed to provide study rationale for specific users’ circumstances such as various condom use experience and relationship status or message strengthening program credibility perception)

Additional pages

- Contact form
- Reminders cancellation
- Login
- Exit
- Password reset

Study pages

- Participant Information Sheet
- Consent statement
- Screening questionnaire
- Registration
- Study questionnaires
- Charity donation
- Debriefing sheet
- Information about uncompleted measures
- Next follow-up date/completion of the study

Condoms and lubricants kit

- Six different types of condoms (2 of each) chosen to give a wide range of sizes, shapes, and materials (latex and non-latex)
- Two types of lubricants in 6 single use sachets
- A printed copy of correct condom use instructions with a link to the study website

## Methods

### Study Design

The study uses a pre-post test, within-subjects design.

### Participants

A target sample of 139 participants was recruited and data collection is completed. The sample size was estimated on the basis of the calculation of the number of participants required to conduct statistical analysis to evaluate the feasibility of the program and its preliminary effectiveness, possible high attrition (in the region of 60%), more likely in self-guided interventions [32-36], study resources, and numbers of participants recruited to similar studies [31,37-42]. The inclusion criteria for the study are listed in Textbox 2.

### Recruitment

Participants were recruited through self-referral in response to recruitment advertisements (posters, leaflets, business card adverts, Facebook and Twitter posts and paid adverts, emails, and United Kingdom-wide mailing lists for postgraduate psychology students). The adverts included key phrases such as “test and rate condoms,” “improve condom use experience,” “learn more about condoms,” “focus on pleasure,” “enjoy using condoms,” and “get free condoms and lubricants kit.” To ensure wide reach and reduce the risk of recruitment bias (age and geographical location) where possible, study advertisements were distributed in multiple locations (mainly in England, including universities, colleges, sexual health charities, commercial sector employers, community centers, youth organizations), and on social media (Facebook paid posts addressed to specific age groups, for example 26 to 35 years and 36 to 45 years with the United Kingdom chosen as geographical location). People from professional and personal networks were also asked to share the advertisements in their locations and through social media.

### Data Collection

Questionnaires and website usage data were used in the study to collect data. The questionnaires were chosen to mirror as closely as possible the measures used in the face-to-face KIHIS [22,26] and HIS-UK studies [27]. They were reviewed and modified according to the feedback received in a qualitative evaluation of the program during its development phase (M Glowacka, thesis chapter in preparation). Additional measures and items were chosen or developed for this study to allow investigation of the aspects of the program related to its specific mode of delivery. The data collection schedule is presented in Table 1.

### Measures

#### Eligibility Screening Questionnaire

The eligibility screening questionnaire included questions assessing the inclusion and exclusion criteria (see Textbox 2).

#### Registration

Following screening, eligible participants were asked to provide an email address that the study reminders are sent to and an optional phone number if they would also like to receive text messages with study reminders.

#### Background Information Questionnaire

At baseline (T1), participants provided background information such as ethnic background, education, employment, relationship status, first part of the postcode, and computer use proficiency. The ethnic background question categories were adapted from the Census for England [43].

#### Sexual History

At T1, participants were asked about their current sexual activity, where they chose an answer from the following options: (1) sex with one partner only, (2) frequent sex with different partners, (3) infrequent sex with different partners, (4) occasional sex with different partners, (5) not sexually active, and (6) other. 

They were also asked about the gender of their sexual partners (women, men, women and men, I have never had sex) [44] and the number of sexual partners.

#### Sexually Transmitted Infections and Unplanned Pregnancy

At T1, questions about lifetime and last year STI diagnoses and unplanned pregnancies were asked. At T2 and T3 participants provided information about STI diagnoses and unplanned pregnancies in the last 4 weeks.

#### Condom Use and Sexual Activity

To assess the frequency and consistency of condom use at T1 to T3 participants were asked about the number of episodes of penile-vaginal, penile-anal, or penile-oral intercourse in the last 4 weeks, the number of partners in the last 4 weeks, the number of times a condom was used during penile-vaginal, penile-anal, or penile-oral intercourse in the last 4 weeks, and whether they practiced using condoms in the last 4 weeks. Participants also provided reasons for using condoms (I did not use condoms, to avoid STIs, to avoid HIV/acquired immunodeficiency syndrome [AIDS], to please my partner, to make sex more pleasurable, to make sex last longer, so my partner would not get pregnant, to practice, other) and the type(s) of condoms used in the last 4 weeks (latex, non-latex, I don’t know what kind we used, not applicable [I did not use condoms]). In addition, at T1 they were asked whether they were taught how to use condoms, and if so, where they learned to use condoms from (leaflet attached to the condom pack, leaflet given to me, watching condom use demonstration [video], watching condom use demonstration [live], practicing how to use condoms correctly instructed by somebody else [ie, during sex education/in the clinic etc], erotic/porn movie, erotic/porn magazine, have not learnt how to use condoms), and whether they had ever used condoms or practiced using them without a partner present.

#### Condom Use Experience

This questionnaire was only displayed to those who reported that they had used condoms during sexual intercourse over the last 4 weeks (T1, T2, and T3). The Effect on Sexual Experience subscale from the Condom Barriers Scale [45,46] is a 7-item scale which measures participants’ condom use experience at T1 to T3, including condom fit-and-feel, condom mood interruption, and condom impact on climax or orgasm and on the relationship with sexual partner. Items are rated on a 5-point scale from 1 (strongly agree) to 5 (strongly disagree). Higher scores indicate better condom use experience. In previous research this subscale showed good internal reliability with alpha values of .74 [22] and .81 [26].

#### Condom Attitudes

Five items chosen from the Multidimensional Condom Attitudes Scale (MCAS) [47] assessing pleasure associated with condoms were used to assess attitudes toward condoms at T1 to T3 [26]. Items are rated on a 7-point scale from 1 (strongly disagree) to 7 (strongly agree), with higher scores indicating more positive condom use attitude (3 items are reverse scored). An option of “neither agree nor disagree” for the 4th item was added because of participants’ feedback in the qualitative study evaluation eHIS program website (M Glowacka, thesis chapter in preparation). The subscale showed good reliability in the previous study evaluating the KIHIS program [26], with an alpha value of .81.

**Inclusion and exclusion criteria.**

Criteria

- Inclusion
  - Male
  - Aged 18 to 69 years
  - Fluent in English (written and spoken)
  - Have access to the Internet for the duration of the study
  - Living in the United Kingdom
- Exclusion
  - Other than male
  - Below the age of 18 or aged 70 or above
  - Not fluent in English (written and spoken)
  - Allergic or sensitive to latex, non-latex condoms, and/or lubricants
  - Have difficulties using computers and other visual display units equipment requiring use of specialist software to access the website content
  - Have a learning disability requiring third person support to access and use the eHIS website
  - Do not have access to the Internet for the duration of the study
  - Living outside of the United Kingdom

**Table 1:** Schedule of study measures.

Measure	T1	T2	T3
Eligibility screening questionnaire	Yes		
Study registration	Yes		
Motivation to take part in the study	Yes		
Recruitment information	Yes		
Background information	Yes		
Sexual history	Yes		
STIs^a^ and unplanned pregnancy^b^	Yes	Yes	Yes
Condom use and sexual activity^c^	Yes	Yes	Yes
Effect on Sexual Experience subscale from Condom Barriers Scale^d^	Yes	Yes	Yes
Correct Condom Use Self-Efficacy Scale (CCUSS)	Yes	Yes	Yes
Condom Use Errors and Problems Survey (M-CUES)^d^	Yes	Yes	Yes
Condom Fit and Feel Scale^e^	Yes	Yes	Yes
Multidimensional Condom Attitudes Scale (MCAS), selected 5 items	Yes	Yes	Yes
eHIS Evaluation Survey		Yes	
Searching for Condom Use Related Information		Yes	
Condom Rating Form (maximum 15 entries)	Yes^f^	Yes	
Website usage data are collected throughout the period when the website is available to the participants	Yes^f^	Yes	

^a^STI: sexually transmitted infection.

^b^At T1 questions are asked about lifetime and last year, at T2 and T3 about the last 4 weeks.

^c^Additional questions asked at T1 (see measures descriptions).

^d^Questionnaires displayed only to those who reported that they had used condoms during sexual intercourse over the last 4 weeks.

^e^Questionnaires displayed only to those who reported that they had used condoms during sexual intercourse or had practiced using condoms over the last 4 weeks.

^f^Between T1 and T2.

#### Condom Use Self-Efficacy

At T1 to T3 participants’ perception of their condom use ability (eg, finding condoms that fit properly, keeping condoms from drying out during sex) were measured by 7 items adapted from the Correct Condom Use Self-Efficacy Scale (CCUSS) [22,48]. These items are rated on a 5-point scale from 1 (very difficult) to 5 (very easy). Higher scores indicate greater correct condom use self-efficacy, which is associated with fewer condom use errors and problems [49]. This scale was demonstrated to have good internal reliability in previous studies with alpha values of .72 [22], .70 [49], and .82 [27].

#### Condom Use Errors and Problems

The survey was only displayed to those who reported that they had used condoms during sexual intercourse over the last 4 weeks (T1, T2, and T3). The 17-item Condom Use Errors/Problems Survey (M-CUES) [50] assesses condom use errors and problems experienced during the last condom-protected sexual event. Respondents were asked about the presence or absence (yes/no) of problems and errors such as condom breakage and slippage, issues with fit-and-feel, incomplete or incorrect use of condoms, and loss of erection associated with condom use. Separate condom use error and problems scores are calculated, with higher scores indicating more condom use errors and problems. The CUES has good face and content validity [50].

The CUES was modified in line with feedback received from participants in the qualitative study evaluating the eHIS website (M Glowacka, thesis chapter in preparation) and from materials developed for the HIS-UK feasibility study [27]. The form of the questionnaire was simplified, as was the scale instruction and item wording. An item asking about checking a condom expiry date was added to the scale to make it consistent with the condom use instructions given in the program. To make the recollection of events easier the recall time was changed from “last 3 times the condom was used” to “last time you used a condom.”

#### Condom Fit and Feel Scale

The Condom Fit and Feel questionnaire [51] was only displayed to those who reported that they had used condoms during sexual intercourse or practiced condom use over the last 4 weeks (T1, T2, T3). This 14-item scale was completed at T1 to T3. Items include “Condoms fit my penis just fine” and “Condoms are too long for my penis”. Answers are given on a 4-point scale from 1 (never applies to me) to 4 (always applies to me) with some items being reverse scored. An overall score is obtained where higher scores indicate more negative experiences with condom fit-and-feel. Scale validity and reliability have been demonstrated previously, with alpha values ranging from .60 to .86 [52].

#### Condom Rating Form

Participants were asked to complete this form after each condom use practice. In the first part of the form they gave information about which condom they used during a practice session and whether they had used it before. They indicated what type of sexual activity the condom was used for, whether they stopped testing it before putting it on, and if yes, what was the reason. In the second part of the rating form participants rated condoms on different aspects of fit-and-feel. They were also asked about the use of lubricant and their preference for using the particular condom in the future. Participants were expected to complete at least 6 condom rating forms; a maximum of 15 ratings could be completed across the time when participants had access to the program’s website. The condom rating form was adapted from materials used in previous studies evaluating the face-to-face version of the program [22,26,27] and modified in line with feedback received in the qualitative evaluation of the program’s computerized version (M Glowacka, thesis chapter in preparation).

#### eHIS Evaluation Survey

The eHIS Evaluation Survey assessed the acceptability of the program’s content and format at the first follow-up (T2). It was developed for this study to explore participants’ opinions about the program and the website. A literature search of previous studies using questionnaires to evaluate electronic health (eHealth) interventions, treatment preferences, and measures used to evaluate websites’ content and usability [42,53-60] and themes identified in the qualitative phase of the eHIS website development (M Glowacka, thesis chapter in preparation) were used to define key categories and guided item development.

The 24-item survey assessed agreement or disagreement (from strongly disagree to strongly agree) with statements related to relevance of the program for the issues covered, personal relevance, completeness of the information and advice given, willingness to follow the advice given, trustworthiness, clarity of the content, program use enjoyment, website usability, including questions about its structure, navigation, information, organization, and website aesthetics. Participants also had a chance to share their preferences regarding the program’s content and design in open text entry questions, as well as provide additional qualitative feedback. For the item “The amount of the information on the page was…” the responses were “just right”, “too much”, and “not enough”.

#### Searching for Condom Use-Related Information

This is a 3-item questionnaire developed for this study that was completed at T2 only. Participants were asked whether they searched for additional condom use information when they had access to the eHIS website and if yes, where they searched for the information (social media, National Health Service website, other health information websites, sexual health clinic, general practitioner surgery, youth center, friends, other) and what type of information it was (correct condom use instruction, advice on dealing with condom use problems, information about different types of condoms, information about different types of lubricants, other). Answers to these questions, together with the answers from the eHIS evaluation survey, will be used to assess the program's completeness and credibility (dimensions of acceptability).

Website usage data is also used as a measure of participants’ engagement with the program [31,35,39,61]; the eHIS website logs are used to analyze participants’ activities on the website including time spent on the website, number of visits, and specific pages seen by participants.

Whether participants ordered the condoms and lubricants kit and the number of completed condom rating forms are used as measures of engagement with the program alongside participants’ self-reports on the specific items in the eHIS evaluation survey.

The feasibility of the study evaluation approach is also assessed in the context of the recruitment information, motivation to take part in the study, specific outcome measures completion, and attrition rate. At T1, participants were asked how they heard about the study, what were their reasons for taking part (a multiple choice question), and whether they took part in any study at the program’s development stage. Measures' acceptance is assessed on the basis of proportion of participants completing specific scales and providing answers to their specific items. Attrition is assessed on the basis of completion rate of baseline and follow-up questionnaires.

### Study Procedure

Following the link or QR code from the advertisement individuals were directed to the study website where they were first presented with the Participant Study Information Sheet. Participants indicated their consent to take part and for their data to be used for research purposes by ticking a box next to the consent statement. They then completed the eligibility screening measure; if eligible, they were directed to the study registration page and then to the T1 measures. If ineligible, they were thanked for their interest in the study.

Participants could then access the core eHIS website and were able to order a condom kit to be sent by post or collected from the University of Southampton within 3 working days from placing the order. They had 4 weeks, counting from the date they completed the T1 measures, (hereafter “start point”) to practice condom use at home and complete condom rating forms after each practice event. Four weeks from the start point the website was no longer available to participants and at that point participants were asked to complete T2 measures; 10 weeks after the start point they were asked to complete the final T3 measures.

**Figure 2:**
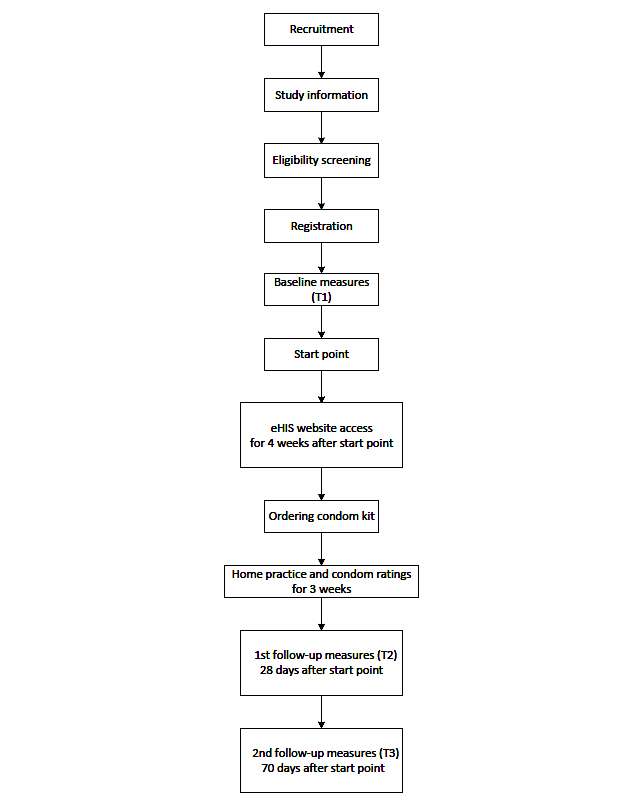
Study procedure.

Participants received an email reminder and an optional text reminder on the days the T2 and T3 measures were due to be completed. They also received 2 emails and an optional text per week for the duration of home practice (during weeks 2, 3, and 4) reminding them to complete condom ratings. The condom rating reminders were automatically cancelled for the particular week if at least 1 rating was completed; all reminders were automatically cancelled if at least 4 ratings were completed. Participants had the option to cancel emails and/or text messages when they visited the program’s website regardless of the number of ratings reminders. An overview of the study procedure is presented in Figure 2. Ethical approval from the Department of Psychology Ethics Committee at the University of Southampton was obtained. The study is registered in the Research Registry, Unique Identifying Number researchregistry2325.

### Incentives

After completion of each set of study measures participants chose 1 out of 3 charities that will receive a 50p donation. After completion of the T3 measures participants received a £5 Amazon voucher. Psychology students at the University of Southampton had the option to claim up to 32 research credits for participation.

### Data Analysis

Feasibility of the program and the evaluation approach will be assessed through the analysis of program engagement, acceptability, recruitment, and retention rates. Descriptive statistics will be used to describe the study population, feasibility of the evaluation approach, engagement with the program, and its acceptability. The preliminary effectiveness of the program will be assessed through evaluation of the change on primary and secondary condom use-related outcomes. Within group comparison will be undertaken to assess whether there are any differences between specific subgroups (eg, those who complete the study and those who drop out, those reporting improvement on various dimensions of condom use, and those who do not report change) on characteristics such as demographic variables, sexual history, and/or baseline condom use-related variables where sufficient data will be available. The results of the preliminary effectiveness results will be used to calculate the effect size of changes in condom use related outcomes. SPSS software v.21.0 [62] will be used for data analysis.

## Results

Recruitment for the study is complete and data collection was complete in July 2017. Data analysis is in progress and is expected to be completed by February 2018.

## Discussion

It is expected that this study will provide preliminary information about the feasibility of eHIS, specifically engagement with the program, its acceptability and potential to improve condom use frequency and consistency, reduce condom use errors and problems, improve condom use self-efficacy, improve condom use experience, and improve condom use attitudes. These findings can further direct the program’s content and procedure improvements. The results of an exploratory evaluation may support the need for further program development and/or indicate the need for conducting a large scale RCT and provide valuable guidance regarding its optimal design [39,63]. The findings are expected to have scientific and clinical implications, advancing knowledge about the feasibility and preliminary effectiveness of this novel approach to promote consistent and correct condom use and potentially contributing to the development of a tool that could be used in sexual health promotion practice.

## Abbreviations

CUES: Condom Use Errors/Problems Survey
eHIS: Homework Intervention Strategy
KIHIS: Kinsey Institute Homework Intervention Strategy
RCT: randomized controlled trial
STI: sexually transmitted infection
